# Appendicitis Presenting Concurrently with Cecal Arteriovenous Malformation in a Child

**Published:** 2015-09-01

**Authors:** Sahil P Parikh, Eric Rosenberg, Manuel E Portalatin, Elias Fakhoury, Robert V Madlinger

**Affiliations:** St. Joseph’s Regional Medical Center, 703 Main St. Paterson, NJ 07053

**Keywords:** Vascular malformation, Appendicitis, Arterio-venous malformation

## Abstract

Acute appendicitis is a commonly diagnosed surgical problem in the pediatric population. Arterio-venous malformations (AVM) of the colonic tract are rarely reported in the pediatric literature. A 13-year old boy who presented with acute appendicitis with concurrent cecal AVM is reported in whom appendectomy was done. Later on radiological investigations AVM was confirmed.

## CASE REPORT

A 13-year-old male presented with moderate to severe intensity episodes of abdominal pain for 2 months. The pain became localized to the right lower quadrant over a period 3 days. It was associated with fever and chills. He also had frequent episodes of diarrhea. On examination he had pulse of 101, blood pressure 113/75mmHg, and respiratory rate of 20/min. There was tenderness in right iliac region. Routine laboratory tests were within normal limits. CT scan showed a thickened appendix with associated lymphadenopathy. The patient was planned for a laparoscopic appendectomy. Intraoperatively a pulsatile mass was noted on the cecum and proximal ascending colon (Fig. 1). Patient underwent appendectomy. Histopathology of the specimen was consistent with acute appendicitis. The AVM noted intra-operatively was not addressed during the initial procedure due to lack of informed consent, and it was not emergent.

**Figure:**
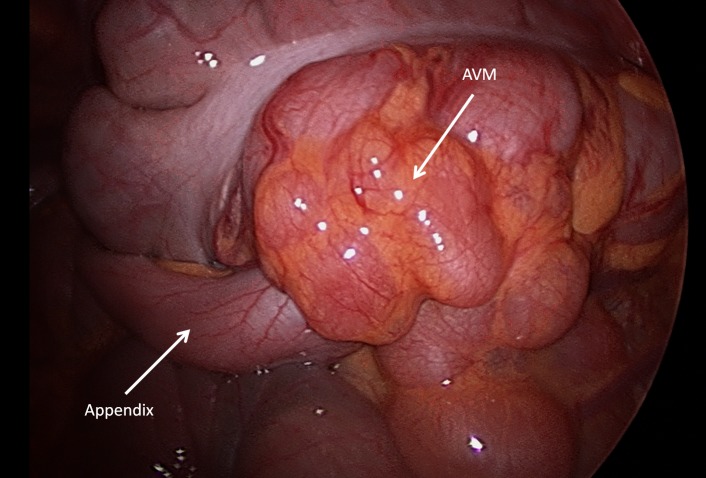
Figure 1:Laparoscopy of the cecum and appendix seen with arteriovenous malformation (AVM).

Postoperatively, patient underwent a CT angiography, which showed simultaneous filling of the ileocolic artery and ileocolic vein, suggesting an AVM (Fig. 2). The patient had an unremarkable postoperative course and was planned for limited right hemicolectomy but parents did not return for the surgical procedure.

**Figure:**
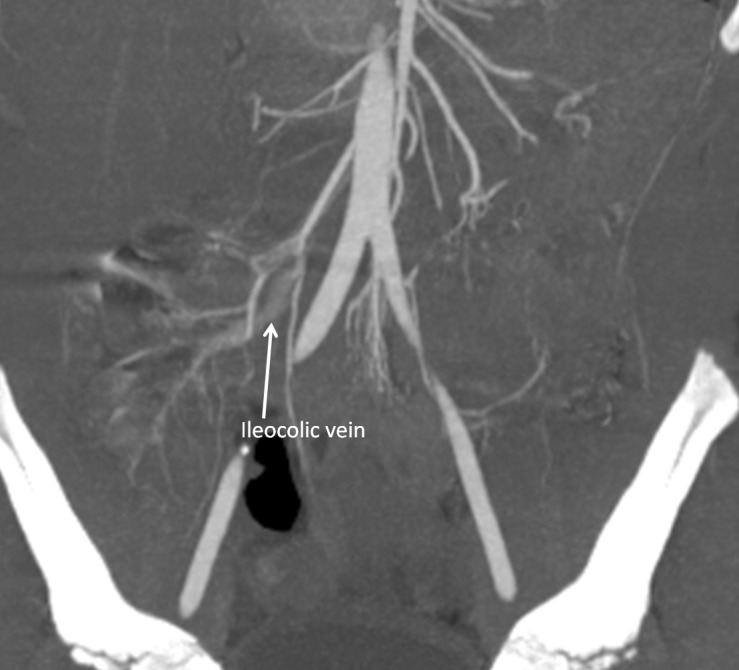
Figure 2:Simultaneous filling of the arterial system and the ileocolic vein.

## DISCUSSION

Acute appendicitis is a common condition in the pediatric population, and the diagnosis of which is usually made on clinical grounds. The need for imaging modalities becomes necessary when clinical signs are not obvious. On the contrary, a diagnosis of colonic AVM is challenging.[1,2] AVMs are due to abnormal connections between veins and arteries, bypassing the capillary bed. The absence of capillaries coupled with shunted blood flow, often results in local tissue ischemia. Additionally, arteriovenous malformations progressively enlarge with time due to an absence of the dampening effect that the capillary beds provide. Ultimately this malformation will form a tangled mass of fragile vessels known as a nidus.[3] The process by which AVMs develop is not clear despite extensive research. A large proportion of AVMs are asymptomatic but they can manifest with a myriad of symptoms and imaging is needed for diagnosis. Delay in diagnosis is common.

Pediatric patients with suspected AVMs may present with intestinal obstruction, diarrhea, pain, emesis, and/or bleeding.[4] A similar case has been reported by Goldstein et al in 2005.[5] In their patient, appendicitis was imitated by an inflammatory reaction and omental adhesions causing fever and tenderness in right iliac region due to a cecal arteriovenous malformation. However, there is no indexed case of an AVM found with a presentation of acute appendicitis.

## Footnotes

**Source of Support:** Nil

**Conflict of Interest:** None declared

